# Supplementary material for: Stochastic Fluctuations of the Facultative Endosymbiont Wolbachia due to Finite Host Population Size
Source: Ecol Evol. 2025 Aug 17;15(8):e71989. doi: 10.1002/ece3.71989 (PMC12358320; doi:10.1002/ece3.71989)
Supplement: Supplementary file 1 — Data S1: ece371989‐sup‐0001‐Supinfo01.docx. [file ECE3-15-e71989-s001.docx]

**Supplemental Information**

**Table S1.** Populations at each stage of host reproduction and development. Here, *N* is the initial female population, *I* is the initial number of *Wolbachia-*positive females, $X_{1}\sim Bin\left( FpI,1-\mu\right)$, and $X_{2}\sim Bin\left( F\left( 1-p \right)I,1-\mu\right).$

|  | ***Wb*^+^ Female**  ***Wb*^+^ Male** | ***Wb*^+^ Female *Wb^‒^* Male** | ***Wb^‒^* Female**  ***Wb*^+^ Male** | ***Wb^‒^* Female**  ***Wb^‒^* Male** |
| --- | --- | --- | --- | --- |
| **Matings** | *pI* | $\left( 1-p \right)I$ | $p\left( N-I \right)$ | $\left( 1-p \right)(N-I)$ |
| **Ova** | *FpI* | $F\left( 1-p \right)I$ | $p\left( N-I \right)$ | $\left( 1-p \right)(N-I)$ |
| **Ova after imperfect transmission** | *X_1_* | *X_2_* | $p\left( N-I \right)+FpI-X_{1}$ | $\left( 1-p \right)\left( N-I \right)+F\left( 1-p \right)I-X_{2}$ |
| **Adult offspring after CI** | *X_1_* | *X_2_* | $\left( 1-s_{h} \right)(p\left( N-1 \right)+FpI-X_{1})$ | $\left( 1-p \right)\left( N-I \right)+F\left( 1-p \right)I-X_{2}$ |

**Table S2.** Regression analysis of the effects of model parameters on mean *Wolbachia* frequencies ($\bar{p}$).

|  | | **Coefficient** | **t value** | ***P* value** |
| --- | --- | --- | --- | --- |
|  | **Host population size** | -1.58 x 10^-8^ | -6.09 | < 0.001 |
| **Imperfect maternal transmission (*µ*)** | ***µ* value (0.001 ≤ *µ* ≤ 0.3)** | -2.24 x 10^1^ | -1.02 x 10^3^ | < 0.001 |
|  | **Low transmitters** | -2.43 | -7.33 x 10^2^ | < 0.001 |
|  | ***µ* value * low transmitters** | 8.26 | 2.76 x 10^2^ | < 0.001 |
| **Host fitness effects (*F*)** | ***F* value (1 ≤ *F* ≤ 1.5)** | 3.77 | 4.01 x 10^2^ | < 0.001 |
|  | **Fluctuating *F* (*CV* = 0.01)** | 1.30 x 10^-4^ | 4.93 x 10^-2^ | 9.61 x 10^-1^ |
|  | **Fluctuating *F* (*CV* = 0.1)** | 9.68 x 10^-3^ | 3.62 | < 0.001 |
| **Cytoplasmic incompatibility (*s_h_*)** | **Weak CI (*s_h_* = 0.1)** | 4.27 x 10^-1^ | 1.63 x 10^2^ | < 0.001 |
|  | **Strong CI (*s_h_* = 0.45)** | 1.92 | 6.82 x 10^2^ | < 0.001 |

**Table S3.** Regression analysis of the effects of model parameters on the standard deviation of *Wolbachia* frequencies (*p*_SD_).

|  | | **Coefficient** | **t value** | ***P* value** |
| --- | --- | --- | --- | --- |
|  | **Host population size** | -4.14 x 10^-7^ | -1.13 x 10^2^ | < 0.001 |
| **Imperfect maternal transmission (*µ*)** | ***µ* value (0.001 ≤ *µ* ≤ 0.3)** | 8.51 | 4.03 x 10^2^ | < 0.001 |
|  | **Low transmitters** | 1.12 | 2.88 x 10^2^ | < 0.001 |
|  | ***µ* value * low transmitters** | -4.42 | -1.39 x 10^2^ | < 0.001 |
| **Host fitness effects (*F*)** | ***F* value (1 ≤ *F* ≤ 1.5)** | -2.04 | -1.68 x 10^2^ | < 0.001 |
|  | **Fluctuating *F* (*CV* = 0.01)** | 4.23 x 10^-1^ | 1.01 x 10^2^ | < 0.001 |
|  | **Fluctuating *F* (*CV* = 0.1)** | 1.78 | 4.49 x 10^2^ | < 0.001 |
| **Cytoplasmic incompatibility (*s_h_*)** | **Weak CI (*s_h_* = 0.1)** | -9.85 x 10^-2^ | -3.20 x 10^1^ | < 0.001 |
|  | **Strong CI (*s_h_* = 0.45)** | -7.83 x 10^-1^ | -2.17 x 10^2^ | < 0.001 |

**
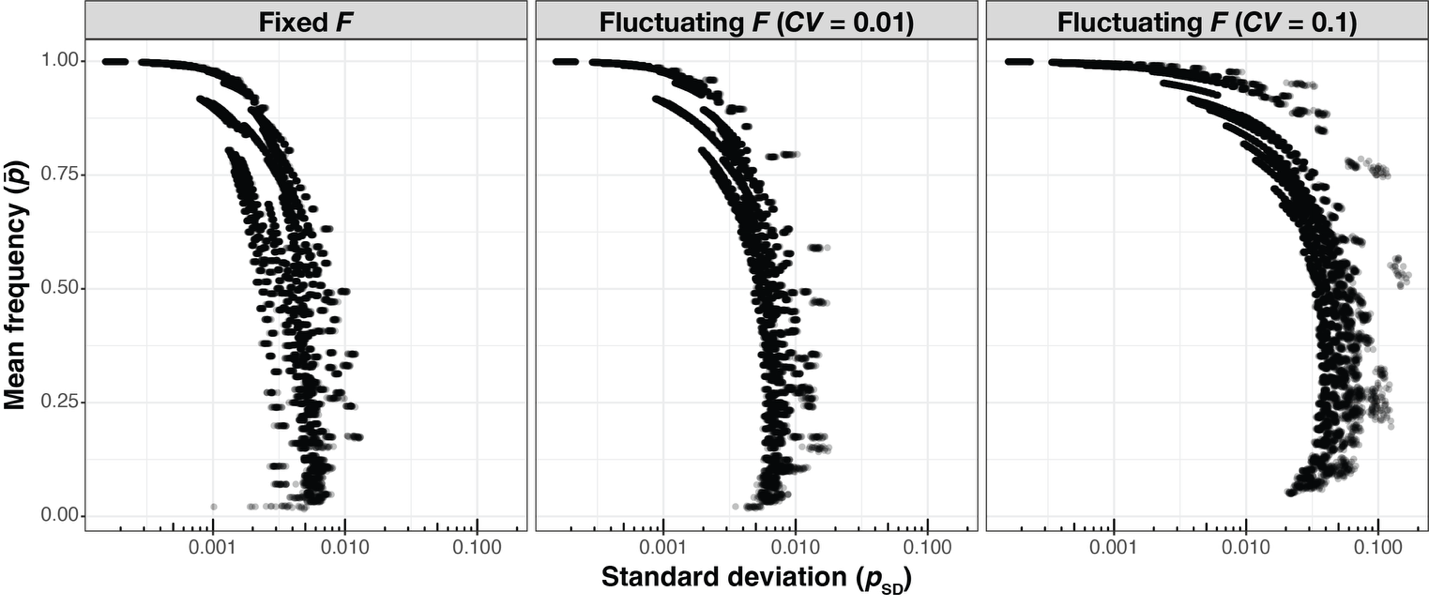
Figure S1.** The relationship between mean infection frequency ($\bar{p}$) and the standard deviation (*p*_SD_) in simulations with a host population of *N* = 10^4^. Each individual point represents the $\bar{p}$ and *p*_SD_ values from a single simulation. Datasets are separated based on whether fluctuating host effects (*CV* = 0.01 or 0.1) are present.

**
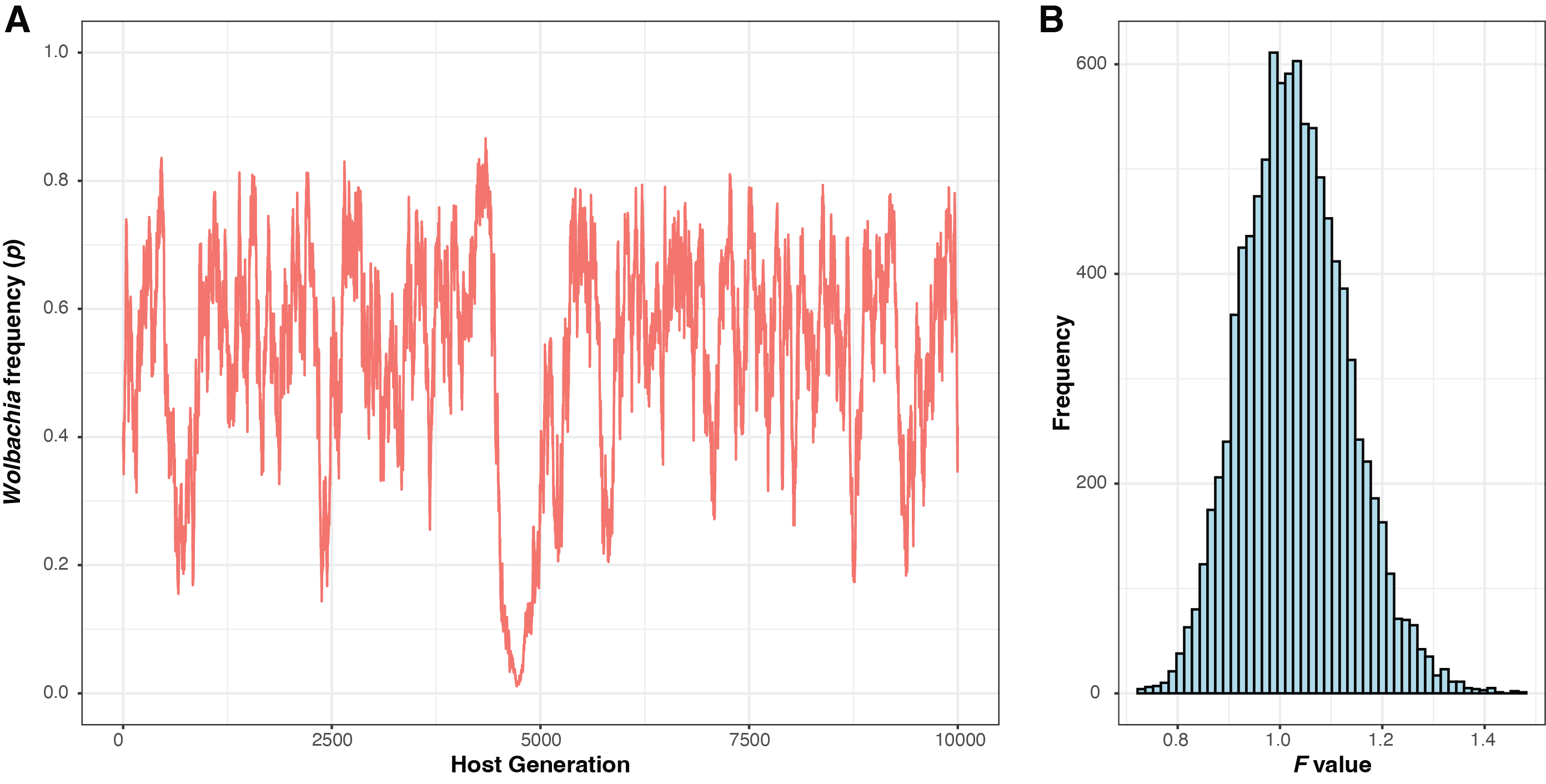
**

**Figure S2. (A)** For a host population size of *N* = 10^4^ and fluctuating host fitness effects, the parameter values of *s_h_* = 0, *F =* 1.025 (*CV* = 0.1), and *μ* = 0.01 (without low transmitters) produced the largest average *p*_SD_ value of $\bar{p_{\mathrm{SD}}}$ = 0.144 across 25 replicate simulations ($p$ = 0.540). One of the 25 simulations is shown here as an example. **(B)** Histogram of *F* values from 10,000 host generations for *F* = 1.025 (*CV* = 0.1). Strongly fluctuating *F* values with a median slightly greater than one cause *Wolbachia* to alternate between favored (*F*[1 – *µ*] > 1) and disfavored (*F*[1 – *µ*] < 1) in the host population due to the large number of host generations with *F* < 1.

**
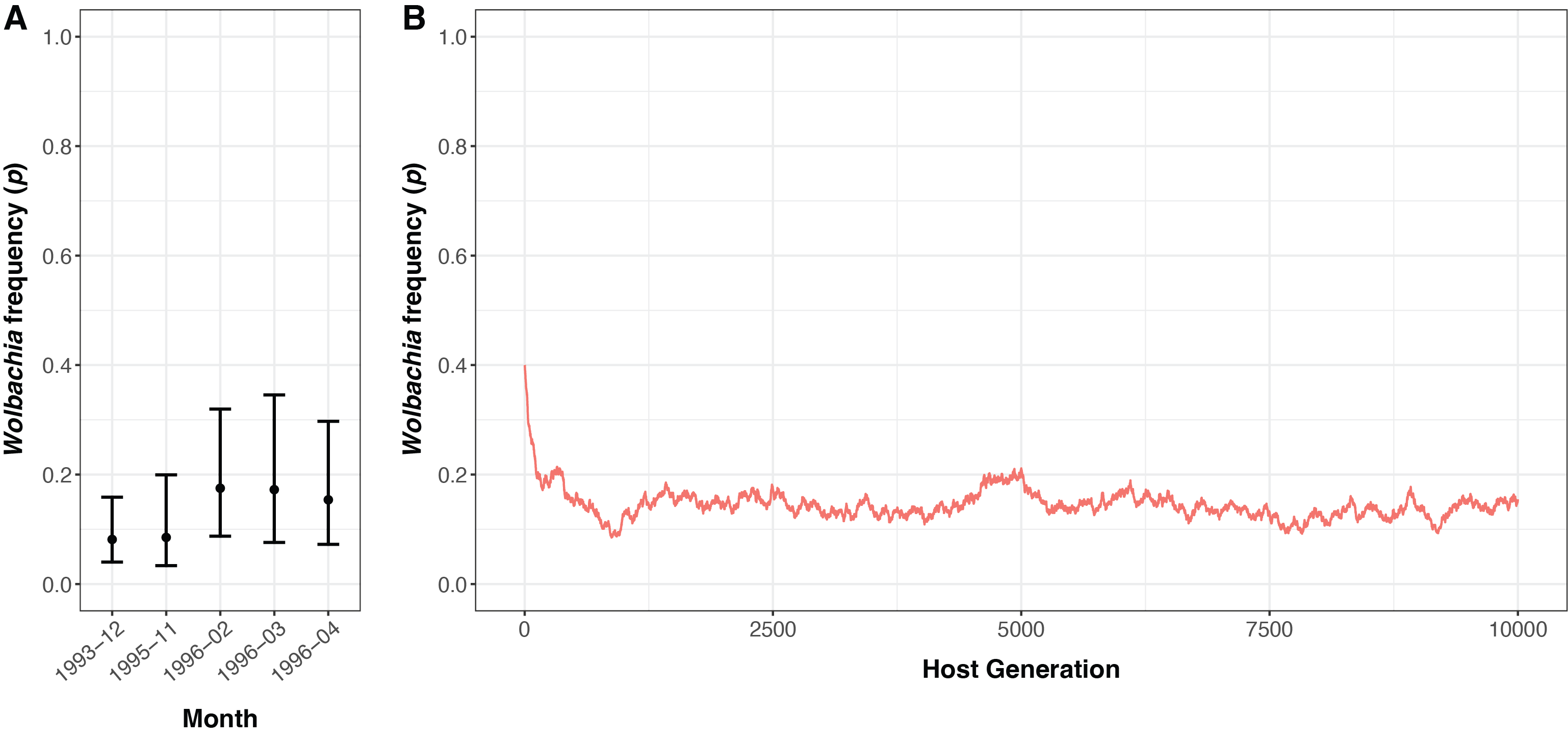
**

**Figure S3. (A)** Temporal fluctuations of *w*Mel frequencies (*p*) in *D. melanogaster* at Hastings in temperate eastern Australia. From the winter of 1993 to the spring of 1996, *p* values exhibited minor fluctuations between a minimum of *p* = 0.081 (0.040, 0.159) and a maximum of *p* = 0.175 (0.087, 0.319). Data recreated from Hoffmann et al. (1998). **(B)** For a host population size of *N* = 10^3^, the parameter values of *s_h_* = 0.1, *F =* 1.1, and *μ* = 0.01 (with low transmitters) produced an average of $p$ = 0.140 and $\bar{p_{\mathrm{SD}}}$ = 0.020 across 25 replicate simulations. One of the replicates is shown here as an example, where *p* values fluctuated between a minimum of 0.085 and maximum of 0.211, which fall within the 95% binomial confidence intervals of the minimum and maximum *w*Mel frequencies observed at Hastings.

**
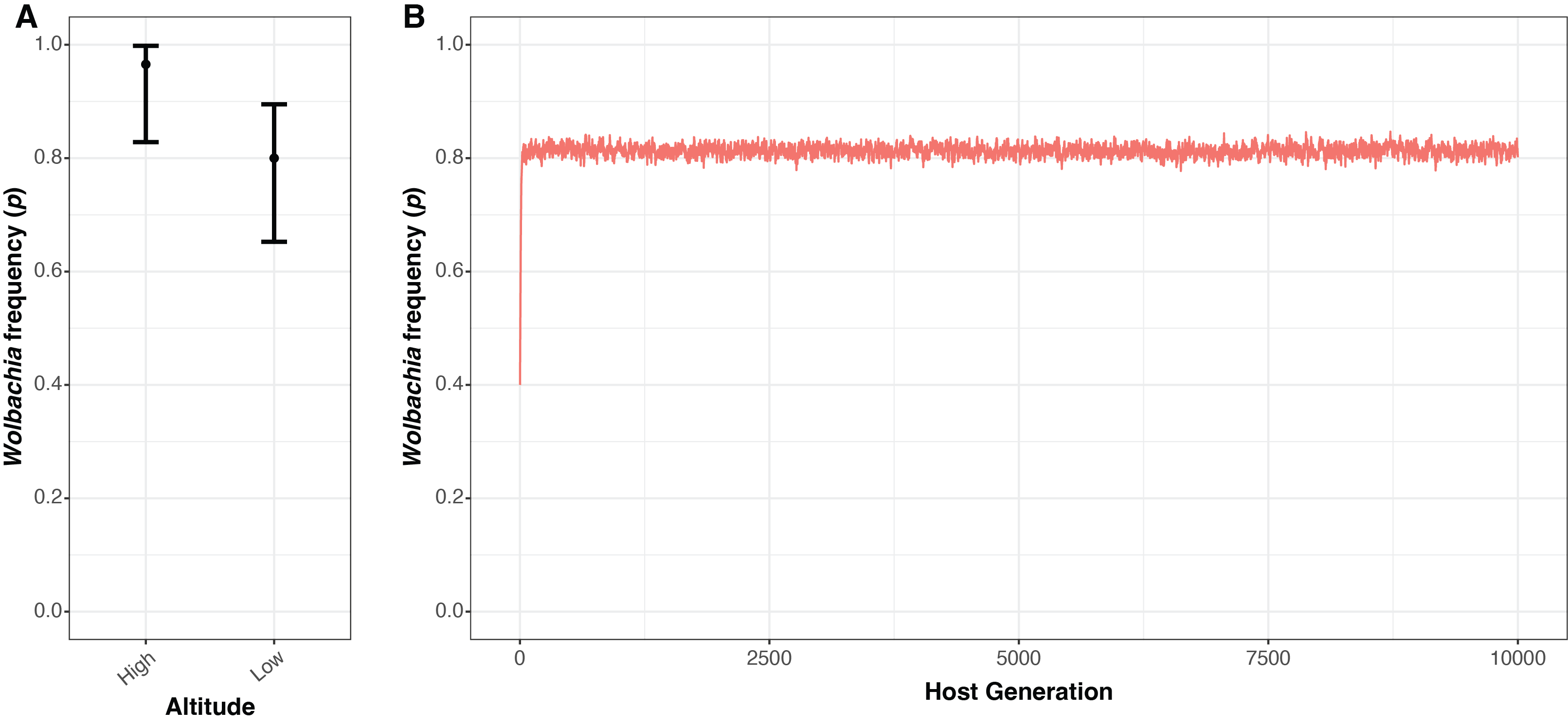
Figure S4. (A)** Altitudinal variation of *w*Yak frequencies (*p*) in *D. yakuba* on the volcanic island of São Tomé off the coast of western Africa. In 2018, *p* values differed between high (*p* = 0.966 [0.828, 0.998]) and low (*p* = 0.800 [0.652, 0.895]) altitude sites on the island. The high and low altitude sites are separated by 310 m of elevation and 2.4 km of distance. Data recreated from Hague et al. (2020). **(B)** For a host population size of *N* = 10^3^, the parameter values of *s_h_* = 0.1, *F =* 1.225, and *μ* = 0.05 (no low transmitters) produced an average of $p$ = 0.813 and $\bar{p_{\mathrm{SD}}}$ = 0.010 across 25 replicate simulations. One of the replicates is shown here as an example, where *p* values fluctuated between a minimum of 0.777 and maximum of 0.847, which fall within the 95% binomial confidence intervals of the high and low altitude *w*Yak frequencies observed at São Tomé in 2018. See Hague et al. (2020) for further details about the contribution of maternal transmission rates to altitudinal *w*Yak frequencies.

**
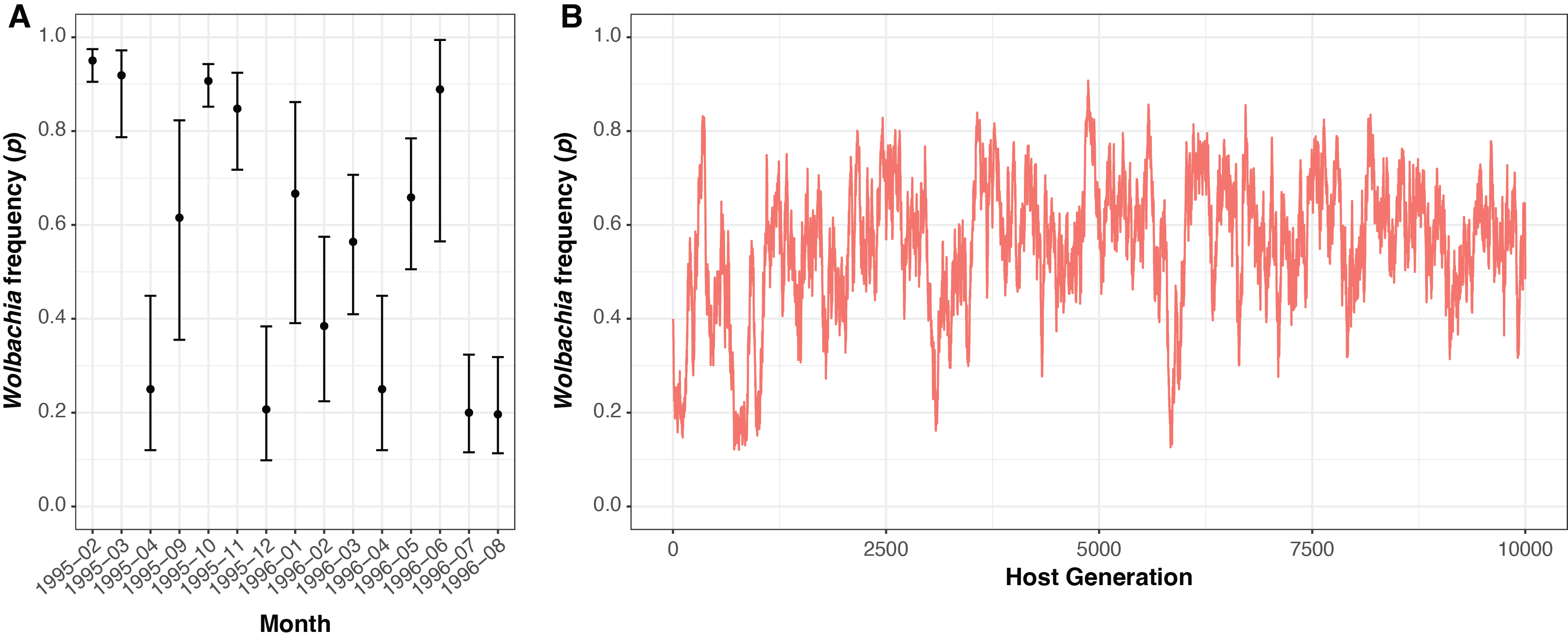
Figure S5. (A)** Temporal fluctuations of *w*Mel frequencies (*p*) in *D. melanogaster* at Gold Coast in subtropical eastern Australia. From the winter of 1995 to the summer of 1996, *p* values fluctuated between a minimum of *p* = 0.196 (0.113, 0.318) and a maximum of *p* = 0.950 (0.905, 0.975). Data recreated from Hoffmann et al. (1998). The authors reported no obvious seasonal effect on *w*Mel frequencies. **(B)** For a host population size of *N* = 10^4^ and fluctuating host fitness effects, the parameter values of *s_h_* = 0, *F =* 1.025 (*CV* = 0.1), and *μ* = 0.01 (no low transmitters) produced an average of $p$ = 0.540 and $\bar{p_{\mathrm{SD}}}$ = 0.144 across 25 replicate simulations. One of the replicates is shown here as an example, where *p* values fluctuated between a minimum of 0.121 and maximum of 0.908, which fall within the 95% binomial confidence intervals of the minimum and maximum *w*Mel frequencies observed at Gold Coast.

**
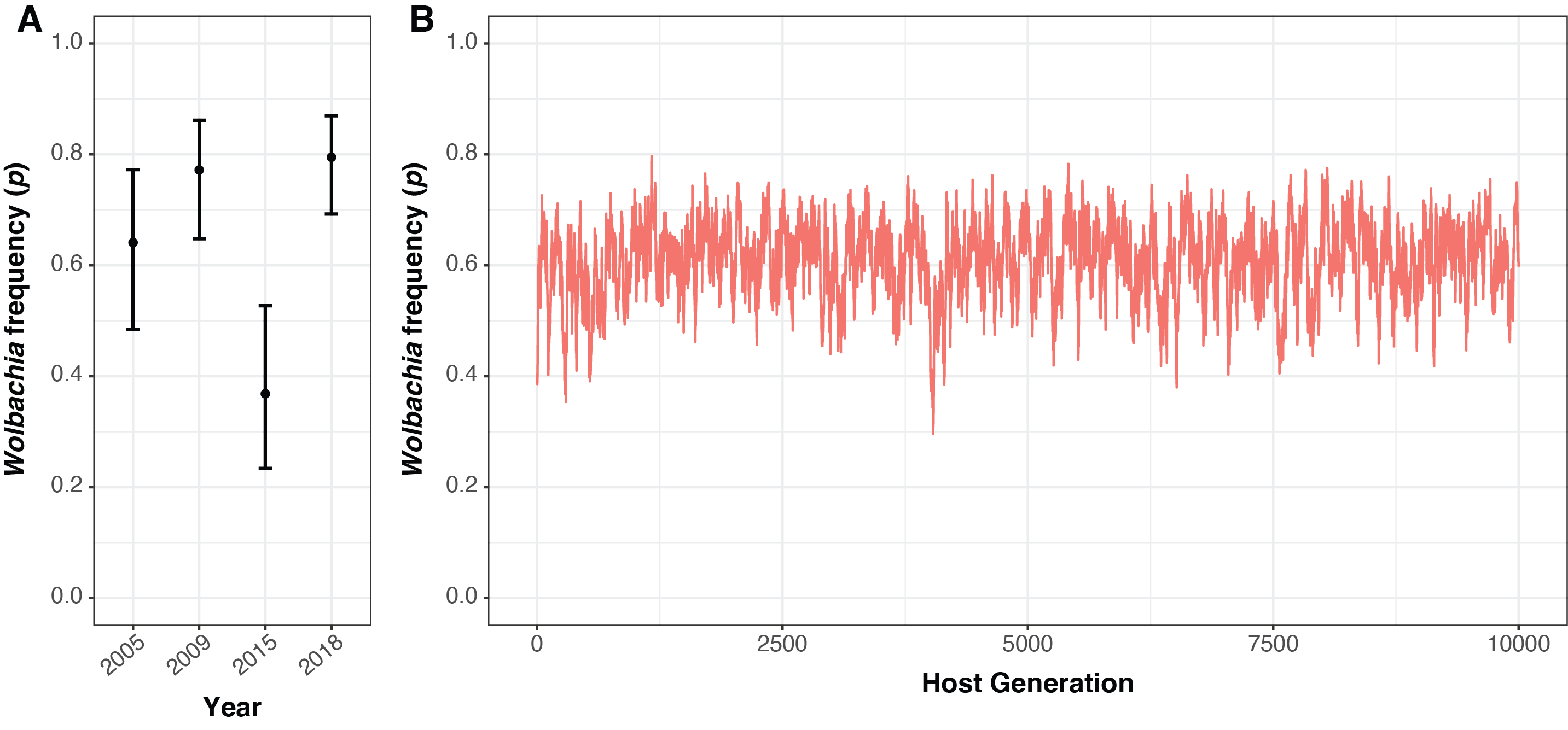
Figure S6.** Temporal fluctuations of *w*San frequencies (*p*) in *D. santomea* on the volcanic island of São Tomé off the coast of western Africa. Over the course of 13 years, *p* values fluctuated between a minimum of *p* = 0.368 (0.234, 0.527) and a maximum of *p* = 0.795 (0.692, 0.870). Data recreated from Hague et al. (2020). **(B)** For a host population size of *N* = 10^4^ and fluctuating host fitness effects, the parameter values of *s_h_* = 0.1, *F =* 1.075 (*CV* = 0.1), and *μ* = 0.05 (no low transmitters) produced an average of $p$ = 0.606 and $\bar{p_{\mathrm{SD}}}$ = 0.070 across 25 replicate simulations. One of the replicates is shown here as an example, where *p* values fluctuated between a minimum of 0.296 and maximum of 0.797, which fall within the 95% binomial confidence intervals of the minimum and maximum *w*San frequencies observed on São Tomé.
